# Association of Hospital Discharge Against Medical Advice With Readmission and In-Hospital Mortality

**DOI:** 10.1001/jamanetworkopen.2020.6009

**Published:** 2020-06-11

**Authors:** Sally Y. Tan, Jeremy Y. Feng, Cara Joyce, Jonathan Fisher, Arash Mostaghimi

**Affiliations:** 1 Harvard Medical School, Boston, Massachusetts; 2 Department of Internal Medicine, Brigham and Women’s Hospital, Boston, Massachusetts; 3 Department of Internal Medicine, Massachusetts General Hospital, Boston; 4 Department of Public Health Sciences, Loyola University Chicago, Chicago, Illinois

## Abstract

**Importance:**

Hospital readmissions contribute to higher expenditures and may sometimes reflect suboptimal patient care. Individuals discharged against medical advice (AMA) are a vulnerable patient population and may have higher risk for readmission.

**Objectives:**

To determine odds of readmission and mortality for patients discharged AMA vs all others, to characterize patient and hospital-level factors associated with readmissions, and to quantify their overall cost burden.

**Design, Setting, and Participants:**

Nationally representative, all-payer cohort study using the 2014 National Readmissions Database. Eligible index admissions were nonobstetrical/newborn hospitalizations for patients 18 years and older discharged between January 2014 and November 2014. Admissions were excluded if there was a missing primary diagnosis, discharge disposition, length of stay, or if the patient died during that hospitalization. Data were analyzed between January 2018 and June 2018.

**Exposures:**

Discharge AMA and non-AMA discharge.

**Main Outcomes and Measures:**

Thirty-day all-cause readmission and in-hospital mortality rate.

**Results:**

There were 19.9 million weighted index admissions, of which 1.5% resulted in an AMA discharge. Within the AMA cohort, 85% were younger than 65 years, 63% were male, 55% had Medicaid or other (including uninsured) coverage, and 39% were in the lowest income quartile. Thirty-day all-cause readmission was 21.0% vs 11.9% for AMA vs non-AMA discharge (*P* < .001), and 30-day in-hospital mortality was 2.5% vs 5.6% (*P* < .001), respectively. Individuals discharged AMA were more likely to be readmitted to a different hospital compared with non-AMA patients (43.0% vs 23.9%; *P* < .001). Of all 30-day readmissions, 19.0% occurred within the first day after AMA discharge vs 6.1% for non-AMA patients (*P* < .001). On multivariable regression, AMA discharge was associated with a 2.01 (95% CI, 1.97-2.05) increased adjusted odds of readmission and a 0.80 (95% CI, 0.74-0.87) decreased adjusted odds of in-hospital mortality compared with non-AMA discharge. Nationwide readmissions after AMA discharge accounted for more than 400 000 inpatient hospitalization days at a total cost of $822 million in 2014.

**Conclusions and Relevance:**

Individuals discharged AMA have higher odds of 30-day readmission at significant cost to the health care system and lower in-hospital mortality rates compared with non-AMA patients. Patients discharged AMA are also more likely to be readmitted to different hospitals and to have earlier bounce-back readmissions, which may reflect dissatisfaction with their initial episode of care.

## Key Points

**Question:**

What are hospital readmission and mortality outcomes after discharge against medical advice?

**Findings:**

In this nationally representative, all-payer cohort study that included nearly 20 million hospital admissions, discharge against medical advice was associated with a 2.01 increased adjusted odds of 30-day all-cause readmission and a 0.80 decreased adjusted odds of 30-day in-hospital mortality compared with non–against medical advice discharge. Readmissions after discharge against medical advice accounted for more than 400 000 inpatient hospitalization days at a total cost of more than $800 million annually.

**Meaning:**

Patients leaving against medical advice face higher odds of readmission; hospitals should consider targeted interventions and risk stratification to identify the highest-risk individuals among this vulnerable population.

## Teaser

This cohort study investigates hospital readmission and mortality outcomes after hospital discharge against medical advice.

## Introduction

Individuals who leave the hospital against medical advice (AMA) are at high risk for readmission. They account for 1% to 2% of all hospital discharges.^1,2,3,4^ Patients leaving the hospital AMA are more often younger and male, have lower household incomes, more likely to be homeless, less likely to have physical comorbidity, and more likely to have mental illness, including alcohol and drug use.^2,3^ Prior studies have shown that patients discharged AMA have higher rates of 30-day all-cause readmission and 30-day to 90-day mortality rates, even after adjusting for clinical and socioeconomic confounders. However, the generalizability of these studies is limited because they have all been single-center studies or focused on a specific patient population (eg, Veterans Health Administration patients).^1,3,4,5,6,7^

In this study, we used a nationally representative, all-payer database of discharges in the United States to determine the odds of readmission and in-hospital mortality after AMA discharge. We also identified patient and hospital factors associated with readmissions and assessed total health care use associated with 30-day readmissions.

## Methods

### Data Source

We conducted a retrospective cohort analysis of hospital readmissions using the 2014 all-payer Nationwide Readmissions Database (NRD) published by the Agency for Healthcare Research and Quality (AHRQ). The unweighted NRD data set captures all discharges at nonfederal public and private hospitals from a sampling of 22 geographically dispersed states that report to the State Inpatient Database. These unweighted raw data are sourced from 2048 hospitals, amounting to 51.2% of the total US population and 49.3% of hospitalizations. The NRD subsequently defines discharge-level weights that are used to estimate the entire universe of discharges, in this case, all-payer, short-term acute care hospital discharges across the United States. These weighted data (eg, number of index admissions and readmissions) are then reported with confidence intervals because they are not directly measured frequencies but rather statistical estimates using sampling weights as defined by the NRD. In total, the 2014 NRD contains approximately 15 million unweighted discharges and approximately 35 million weighted discharges.^8^ The Partners Healthcare institutional review board reviewed and approved this study. Patient consent was waived because this database contained only anonymized patient data.

### Study Cohort

**Figure 1.**
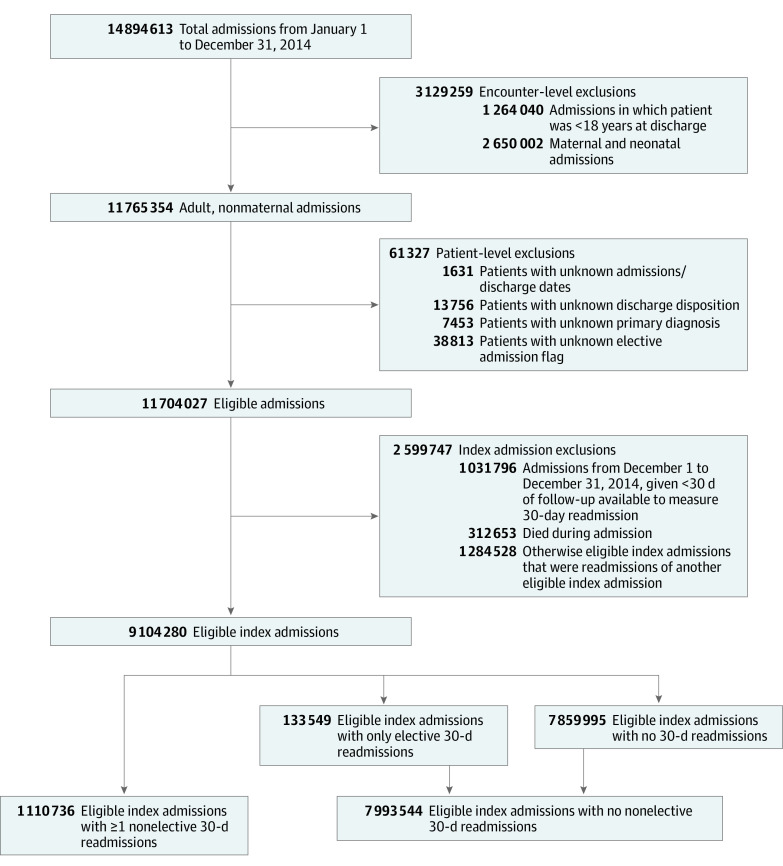
Flowchart of Eligible Index Admissions, Unweighted Flowchart illustrating exclusionary criteria to identify the eligible index admissions evaluated in the study. After applying exclusions, eligible index admissions represented 61% of the total sample set of admissions within the Nationwide Readmissions Database. These numbers represent unweighted frequencies of hospital discharge data reported through the State Inpatient Databases. Standardized weights designed by Healthcare Costs and Utilization Project were applied to obtain weighted estimates of index admissions and readmissions that were nationally representative.

We identified eligible index admissions as detailed in Figure 1. We excluded all hospitalizations for patients younger than 18 years or admissions for obstetrical/newborn care given their high volume and different readmission risk profile vs medical/surgical admissions, as previously reported.^9^ The remaining records only included adult medical (including psychiatric) and surgical inpatient admissions. Patient records with a missing primary diagnosis, discharge disposition, and/or length of stay were also excluded. Finally, discharges that occurred in December, for which 30 days of follow-up were not available, and discharges where the patient died during the hospitalization were excluded as index admissions. The NRD collapses records for multiple hospitalizations involving transfers to an acute care hospital into a single discharge, with subsequent readmissions being attributed to the final discharging hospital. Nonelective admissions for any primary diagnosis occurring within 30 days of the index discharge were deemed readmissions and were not counted as separate index admissions because this would be “double counting” those hospitalizations in our analyses. However, subsequent admissions that occurred at least 30 days after the initial index admission were counted as unique and separate index admissions. Disposition of patient at each discharge is determined from administrative claims and reported as a categorical data element in the NRD, with AMA being a distinct category. Of note, this study excludes AMA discharges from the emergency department because the NRD only reports inpatient admissions.

### Defining Readmissions

We defined a readmission as a nonelective admission for any diagnosis within 30 days of an index admission. We excluded readmissions that were classified as elective in the administrative claim. We included nonelective readmissions for any reason because patients might be admitted for related conditions even if the primary diagnoses on index admission and readmission differ. To calculate the 30-day readmission rate, the numerator was the number of eligible index admissions with at least 1 eligible readmission, and the denominator was the total number of eligible index admissions, as defined in previous paragraphs.

### Patient and Clinical Characteristics

We evaluated associations of readmissions with patient-level (sex, age, insurance status, household income, and comorbid chronic conditions) and hospital-level factors (bed size, teaching status, metropolitan vs rural, and public vs private ownership). Bed size categories are based on the number of hospital beds and are specific to a hospital’s region, urban/rural location, and teaching status. The Chronic Condition Indicator (CCI) was used to dichotomize *International Classification of Diseases, Ninth Revision, Clinical Modification* codes into chronic or nonchronic conditions and to aggregate chronic conditions into 18 mutually exclusive groups as previously described.^10^ All CCI groups were included as independent covariates in our analysis; the total number of CCI groups was also used as a summary indicator of medical complexity.

### Multivariable Regression Modeling

The odds of nonelective readmission by discharge disposition were estimated using logistic regression, both in univariable and multivariable models adjusting for other patient-level and hospital-level characteristics (age, sex, chronic comorbidities, household income, insurance status, hospital size, and teaching status). Covariates were selected a priori based on published literature showing that they were associated with readmissions and/or AMA disposition to control for potential confounders.^1,2,3,4,5,6,7^ We first conducted univariable analyses to estimate associations between each covariate and readmission. Characteristics associated with readmission with *P* less than .20 on univariable analysis were entered simultaneously into a multivariable model. We interpreted both the clinical and statistical significance of differences in our results. Poststratification weights were used, with robust standard errors that appropriately accounted for the survey design of the NRD.^11,12^ Statistical analysis was performed using procedures for survey sampling in SAS, version 9.4 (SAS Institute Inc). A 2-sided *P *value less than .05 was considered significant.

### Sensitivity Analyses

We conducted 2 sensitivity analyses of the 30-day readmission rate. First, we restricted readmissions to only include hospitalizations with the same multilevel Clinical Classifications Software (CCS) diagnosis as the index admission. This increased the likelihood that the second admission was a clinically related readmission. Primary diagnoses were determined using CCS, which is an AHRQ-developed classification system that classifies *International Classification of Diseases, Ninth Revision, Clinical Modification* codes into hierarchical, clinically meaningful groups for statistical analyses. Our results were aggregated at the second level of the multilevel CCS grouping.^13^

Next, we also performed a stratified analysis of 30-day readmission rates comparing a subset of patients with a primary mental health diagnosis on index admission vs all others without. Mental health diagnoses were more prevalent among AMA discharges and may have been a confounder; this was crudely adjusted for using the CCI5 indicator in the baseline multivariable analysis. Patients with primary mental health diagnoses may interact with and receive care by the health care system in meaningfully different ways from all other patients, and there is no consensus about how to account for this in population-level database analyses in the published literature. This sensitivity analysis stratified patients with primary mental health diagnoses using the more specific CCS categorization, in which mental health conditions were defined as CCS groups 600 to 699, which includes alcohol-related disorders (660), substance-related disorders (661), mood disorders (657), schizophrenia and other psychotic disorders (659), and anxiety disorders (651).

### Health Care Utilization Attributable to Readmissions

Total health care utilization associated with readmissions was defined as the sum total length of stay and hospital costs of all subsequent 30-day readmissions attributed to an index admission. Costs were estimated using the AHRQ charge-to-cost ratio as previously described.^14^ Unadjusted 30-day readmission costs and length of stay (LOS) were summarized by median and interquartile ranges given the right-skew distribution of this data. To estimate adjusted costs and LOS for readmissions, we calculated predictive margins from hierarchical γ and negative binomial regressions, respectively, accounting for patient-level and hospital-level covariates as described previously (eg, age, sex, chronic comorbidities, household income, insurance status, hospital size, and teaching status).

## Results

Among 19 882 317 (95% CI, 12 232 775-20 535 955) weighted index admissions, 1.5% (95% CI, 1.4%-1.5%) resulted in an AMA discharge (n = 291 994; 95% CI, 275 044-308 944).

### Readmission and Mortality Rates by Index Disposition

The overall 30-day all-cause readmission rate across all weighted index admissions was 12.1% (95% CI, 11.9%-12.2%). Readmission rates for AMA discharges were 21.0% (95% CI, 20.6%-21.3%) vs 11.9% (95% CI, 11.8%-12.1%) for non-AMA discharges, corresponding to an unadjusted odds ratio (OR) of 1.96 (95% CI, 1.92-2.00; *P* < .001). The overall 30-day in-hospital mortality rate was 5.6% (95% CI, 5.5-5.7%). In-hospital mortality rate for AMA discharges was 2.5% (95% CI, 2.3-2.7%) vs 5.6% (95% CI, 5.5-5.7%) for non-AMA discharges, corresponding to an unadjusted OR of 0.43 (95% CI, 0.40-0.47; *P* < .001) (Table 1).

**Table 1.** Thirty-Day Readmission Rate and Mortality Rate, by Disposition

Index disposition	AMA	Non-AMA	*P* value (AMA vs non-AMA)
30-d All-cause unadjusted readmission rate, % (95% CI)^a^	21.0 (20.6-21.3)	11.9 (11.8-12.1)	<.001
30-d Unadjusted in-hospital mortality rate, % (95% CI)	2.5 (2.3-2.7)	5.6 (5.5-5.7)	<.001
Mean adjusted total 30-d readmission (95% CI)			
Length of stay, d	6.5 (6.4-6.6)	7.3 (7.3-7.4)	<.001
Costs, $	14 643 (14 236-15 050)	15 110 (14 877-15 342)	<.001

Abbreviation: AMA, against medical advice.

^a^ Adjusted mean 30-day total readmission cost and length of stay were estimated as marginal predictions from hierarchical γ and negative binomial regressions, respectively, and adjusting for the patient-level and hospital-level characteristics used in the multivariable analyses.

### Characteristics of AMA Readmissions

**Figure 2.**
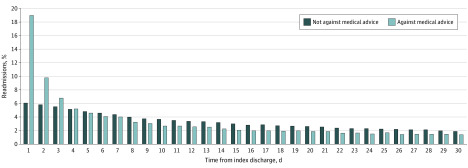
Timing of Readmissions Within 30 Days for Patients Discharged Against Medical Advice (AMA) vs Non-AMA Patients Distribution of the timing of readmissions after AMA vs non-AMA discharge. Data presented reflect the cumulative percentage of 30-day readmissions on each day after initial discharge. Nineteen percent of all 30-day readmissions after AMA discharge occur within the first day after initial hospital discharge, compared with just 6% for non-AMA discharges.

Patients discharged AMA were more likely to be readmitted to a different hospital than their index readmission when compared with all others (43.0% vs 23.9%; *P* < .001). The timing of readmissions also differed between groups because AMA patients were more likely to be readmitted shortly after initial discharge. Of all 30-day readmissions, 19.0% and 6.1% of them occured within the first day after initial discharge for AMA vs non-AMA patients, respectively (Figure 2). Primary diagnosis on readmission also differed between patients initially discharged AMA vs all other dispositions. Notably, diagnoses more common among AMA patients on both index and readmission included alcohol-related disorders, skin and subcutaneous tissue infections, and substance-related disorders (eTables 1 and 2 in the Supplement).

### Characteristics Associated With 30-Day Readmission Rates

Patient-level and hospital-level characteristics differed between AMA vs non-AMA discharge groups. Patients discharged AMA were more likely to be younger, male, have fewer total chronic comorbidities, be in the lowest income quartile, and have Medicaid insurance (Table 2). In univariable analysis of readmissions, middle age (aged 45-64 years), male sex, increased number of chronic comorbidities, Medicare/Medicaid insurance, and discharge from a metropolitan hospital were all associated with higher 30-day readmission rates among patients discharged AMA (Table 3).

**Table 2.** Patient and Hospital Characteristics Among AMA vs All Other Discharge Dispositions

Characteristic	No. of index admissions (% of admissions) [95% CI]	
	AMA	Non-AMA
Age, y		
18-44	126 536 (43.3) [42.4-42.4]	3 632 191 (18.5) [18.1-19.0]
45-64	121 026 (41.4) [40.9-42.0]	6 650 770 (33.9) [33.6-34.3]
>65	44 433 (15.2) [14.6-15.9]	9 307 361 (47.5) [46.8-48.2]
Sex		
Female	108 623 (37.2) [36.6-37.8]	10 418 664 (53.2) [53.0-53.4]
Male	183 371 (62.8) [62.2-63.4]	9 171 659 (46.8) [46.6-47.0]
CCI sum indicator		
0 or 1	64 144 (22.0) [21.7-22.9]	2 419 984 (12.4) [12.0-12.7]
2	59 660 (20.4) [20.0-20.8]	2 785 819 (14.2) [14.0-14.5]
3	57 058 (19.5) [19.2-19.8]	3 626 224 (18.5) [18.3-18.7]
>4	111 133 (38.1) [37.0-39.1]	10 758 295 (54.9) [54.2-55.6]
Median household income quartile		
1st	114 828 (39.3) [37.1-41.6]	5 526 622 (28.2) [26.8-29.6]
2nd	75 482 (25.9) [24.6-27.1]	5 268 502 (26.9) [26.0-27.8]
3rd	53 052 (18.2) [17.1-19.2]	4 453 547 (22.7) [21.9-23.6]
4th	42 658 (14.6) [13.2-16.0]	4 021 218 (20.5) [19.1-21.9]
Expected payer		
Private	43 560 (14.9) [14.1-15.8]	4 854 145 (24.8) [24.2-25.4]
Medicare	87 980 (30.1) [29.4-30.9]	10 486 501 (53.5) [52.9-54.2]
Medicaid	102 328 (35.0) [33.8-36.3]	2 574 796 (13.1) [12.6-13.7]
Other	57 735 (19.8) [18.7-20.9]	1 651 381 (8.4) [8.0-8.9]
Bed size of hospital		
Small	44 606 (15.3) [13.3-17.2]	3 252 274 (16.6) [15.6-17.6]
Medium	90 586 (31.0) [28.1-34.0]	5 406 203 (27.6) [26.2-29.0]
Large	156 802 (53.7) [50.8-56.6]	10 931 845 (55.8) [54.2-57.4]
Hospital teaching status		
Metropolitan		
Teaching	178 181 (61.0) [58.5-63.5]	12 238 239 (62.5) [61.1-63.9]
Nonteaching	89 000 (30.5) [28.2-32.7]	5 385 419 (27.5) [26.3-28.6]
Nonmetropolitan	24 813 (8.5) [7.6-9.4]	1 966 664 (10.0) [9.4-10.7]
Total index admissions, No.	291 994 (275 044-308 944)	19 590 322 (12 232 775-20 535 955)

Abbreviations: AMA, against medical advice; CCI, Chronic Condition Indicator.

**Table 3.** Univariable and Multivariable Logistic Regression Analyses of 30-Day Readmission and In-Hospital Mortality

Characteristic^a^	OR (95% CI)			
	30-d Readmission		30-d In-hospital mortality	
	Unadjusted	Adjusted	Unadjusted	Adjusted
Index admission disposition status				
Non-AMA	1 [Reference]	1 [Reference]	1 [Reference]	1 [Reference]
AMA	1.96 (1.92-2.00)	2.01 (1.97-2.05)	0.43 (0.40-0.47)	0.80 (0.74-0.87)
Age, y				
18-44	1 [Reference]	1 [Reference]	1 [Reference]	1 [Reference]
45-64	1.14 (1.12-1.15)	0.93 (0.92-0.94)	3.03 (2.86-3.22)	2.30 (2.17-2.44)
>65	1.30 (1.28-1.32)	0.83 (0.82-0.85)	6.17 (5.78-6.59)	4.32 (4.05-4.61)
Sex				
Female	1 [Reference]	1 [Reference]	1 [Reference]	1 [Reference]
Male	1.09 (1.08-1.09)	1.03 (1.02-1.04)	1.09 (1.06-1.11)	1.05 (1.03-1.07)
CCI sum indicator				
0 or 1	1 [Reference]^b^^,^^c^	1 [Reference]^b^^,^^c^	1 [Reference]^d^^,^^e^	1 [Reference]^d^^,^^e^
2	1.22 (1.20-1.24)	1.21 (1.19-1.23)	1.68 (1.56-1.82)	1.49 (1.38-1.61)
3	1.33 (1.30-1.36)	1.31 (1.28-1.34)	2.05 (1.89-2.21)	1.73 (1.60-1.86)
>4	1.44 (1.40-1.47)	1.40 (1.28-1.34)	2.38 (2.19-2.58)	1.96 (1.81-2.12)
Median household income quartile				
4th	1 [Reference]	1 [Reference]	1 [Reference]	1 [Reference]
3rd	1.01 (0.99-1.04)	0.99 (0.97-1.01)	0.91 (0.88-0.95)	0.96 (0.93-0.99)
2nd	1.07 (1.04-1.09)	1.02 (1.00-1.04)	0.88 (0.84-0.91)	0.94 (0.91-0.98)
1st	1.18 (1.15-1.21)	1.08 (1.06-1.11)	0.79 (0.75-0.82)	0.91 (0.87-0.95)
Expected payer				
Private	1 [Reference]	1 [Reference]	1 [Reference]	1 [Reference]
Medicare	1.90 (1.86-1.93)	1.70 (1.67-1.73)	1.67 (1.62-1.73)	1.01 (0.98-1.04)
Medicaid	1.97 (1.92-2.02)	1.74 (1.71-1.78)	0.69 (0.66-0.73)	0.93 (0.89-0.97)
Other	1.14 (1.11-1.18)	1.14 (1.11-1.16)	0.65 (0.61-0.70)	0.87 (0.82-0.93)
Bed size of hospital				
Small	1 [Reference]	1 [Reference]	1 [Reference]	1 [Reference]
Medium	1.08 (1.03-1.12)	1.06 (1.02-1.09)	0.97 (0.91-1.04)	1.00 (0.95-1.06)
Large	1.12 (1.07-1.17)	1.08 (1.05-1.11)	0.95 (0.90-1.00)	0.98 (0.93-1.03)
Hospital teaching status				
Nonmetropolitan	1 [Reference]	1 [Reference]	1 [Reference]	1 [Reference]
Metropolitan				
Teaching	1.15 (1.11-1.18)	1.16 (1.13-1.20)	0.87 (0.82-0.92)	0.85 (0.80-0.90)
Nonteaching	1.16 (1.12-1.20)	1.17 (1.13-1.21)	0.86 (0.81-0.91)	0.84 (0.80-0.89)

Abbreviations: AMA, against medical advice; CCI, Chronic Condition Indicator; OR, odds ratio.

^a^ Hospital ownership was excluded from multivariable model because it did not reach statistical significance (*P* < .05) on bivariate screening.

^b^ Each of the 18 CCIs were also included as independent covariates in the bivariate regression on 30-day readmission rates. All of the CCI categories reached statistical significance at *P* < .01 except for CCI3 (endocrine, nutritional, and metabolic diseases; *P* = .46) and CCI15 (perinatal conditions; *P* = .12).

^c^ Each of the 18 CCIs were also included as independent covariates in the multivariable regression on 30-day readmission rates. All of the CCI categories reached statistical significance at *P* < .01 except for CCI15 (perinatal conditions; *P* = .33).

^d^ Each of the 18 CCIs were also included as independent covariates in the bivariate regression on 30-day in-hospital mortality. All of the CCI categories reached statistical significance at *P* < .01 except for CCI1 (infectious diseases; *P* = .89) and CCI3 (endocrine, nutritional, and metabolic diseases; *P* = .12).

^e^ Each of the 18 CCIs were also included as independent covariates in the multivariable regression on 30-day in-hospital mortality. All of the CCI categories reached statistical significance at *P* < .01 except for CCI14 (congenital anomalies; *P* = .69), CCI16 (symptoms, signs, and ill-defined conditions; *P* = .91), CCI17 (injury and poisoning; *P* = .09), and CCI18 (factors influencing health access; *P* = .06).

After adjusting for patient-level and hospital-level characteristics, AMA discharge was associated with higher odds of 30-day readmission (adjusted OR, 2.01; 95% CI, 1.97-2.05) vs non-AMA discharge. Younger age, male sex, having more chronic comorbidities, being in the lowest household income quartile, Medicare/Medicaid insurance enrollment, and discharge from large or metropolitan hospitals were also associated with higher odds of 30-day readmission in the multivariable model (Table 3).

### Sensitivity Analyses

These results remained robust in several sensitivity analyses. After restricting readmissions to only include hospitalizations with the same primary diagnosis as the index admission, the 30-day readmission rate was 9.1% (95% CI, 8.9-9.3) for AMA vs 3.4% (95% CI, 3.4-3.5) for all non-AMA index admissions. This represents an increase in the ratio of readmissions for AMA vs non-AMA discharge from approximately 2-fold in the baseline scenario to 3-fold in this sensitivity analysis. In a stratified multivariable regression model among patients without primary mental health diagnoses (CCS 600-699), AMA discharge remained an independent factor significantly associated with 30-day readmissions (adjusted OR, 2.13; 95% CI, 2.09-2.17). For the subset of patients with a primary mental health diagnosis, AMA discharge was still associated with higher readmission rates (adjusted OR, 1.47; 95% CI, 1.39-1.55), although this measure of association was weaker.

### Characteristics Associated With 30-Day In-Hospital Mortality Rates

In univariable analysis of 30-day in-hospital mortality rates, AMA discharge was associated with a lower odds of mortality (OR, 0.43; 95% CI, 0.40-0.47). After adjusting for patient and hospital characteristics, odds of 30-day in-hospital mortality were still lower for patients discharged AMA vs non-AMA (adjusted OR, 0.80; 95% CI, 0.74-0.87). Increased age, male sex, and having more chronic comorbidities were independently associated with higher in-hospital mortality. Conversely, higher median income quartile, having Medicaid or other insurance (including being uninsured), and discharge from a metropolitan hospital were associated with lower in-hospital mortality (Table 3).

### Inpatient Use and Costs Associated With 30-Day Readmissions

In an analysis of adjusted LOS and health care costs, the mean adjusted 30-day readmission LOS was 6.5 days (95% CI, 6.4-6.6) for patients discharged AMA vs 7.3 days (95% CI, 7.3-7.4) for all others. The mean adjusted 30-day readmission cost was $14 643 (95% CI, $14 236-$15 050) for patients discharged AMA vs $15 110 (95% CI, $14 877-$15 342) for all others (Table 1). Nationally, 30-day readmissions after AMA discharge accounted for 403 264 (95% CI, 376 732-429 796) inpatient hospitalization days at a total cost of $822 million (95% CI, $770-$874 million) in 2014.

## Discussion

In this nationally representative sample of hospital discharges, approximately 1.5% of index admissions resulted in an AMA discharge. After adjusting for patient-level and hospital-level characteristics, patients discharged AMA had 2.01 (95% CI, 1.97-2.05) times the odds of having a 30-day readmission and 0.80 (95% CI, 0.74-0.87) times the odds of 30-day in-hospital mortality compared with all other patients. Patients discharged AMA were also more likely to be readmitted earlier and to a different hospital than their initial admission. In sum, national 30-day readmissions after AMA discharge accounted for more than 400 000 inpatient hospital days at a total cost in excess of $800 million in 2014.

These results build on prior studies showing that patients discharged AMA face higher risk of readmission.^1,3,4,5,6,7^ Our estimated 2.01 increased adjusted odds of 30-day readmission after AMA discharge is in line with previously reported ORs of 1.35 to 2.50.^1,3,4,5,7^ Even after adjusting for age and other confounders, our data showed a 20% decrease in adjusted 30-day in-hospital mortality for patients discharged AMA vs all others. This contradicts prior estimates suggesting a 2-fold increased overall mortality for AMA patients during this period. This discrepency could reflect methodologic differences because prior reports all looked at total mortality rates from public death records (eg, social security index and vital statistics) that more accurately capture all deaths.^1,3^ We believe that in-hospital mortality likely underestimates total mortality rates disproportionately for patients discharged AMA because they are more likely to be homeless, face barriers to accessing care, and die outside of the hospital.^6^

Readmission characteristics differed significantly between patients discharged AMA vs those who were not. Alcohol and substance-related disorders, skin and subcutaneous tissue infections, and nondiabetic pancreatic disease were more common readmission diagnoses after AMA discharge. Patients admitted for some of these diagnoses are more likely to leave AMA during their initial admission and are subsequently more likely to be readmitted for the same issues.^2^ In our sensitivity analysis stratifying patients with and without primary mental health diagnoses, having a mental health disease conferred a protective effect against readmission, a result that was also seen in a study by Garland et al.^8^

Even among patients discharged AMA, those with mental health comorbidities face additional barriers in seeking, accessing, and appropriately following up with care, which may explain their fewer attributable readmissions. Patients were also more likely to be readmitted to a different hospital following AMA discharge, which increases the chances for uncoordinated care, medical errors, and redundant workups.^15^ Finally, readmissions after AMA discharge occurred earlier than for non-AMA patients, with nearly 20% of 30-day readmissions occurring within 1 day of initial discharge. These bounce-back readmissions to different hospitals likely represent inadequate initial treatment of the medical condition and reflect patient dissatisfaction with their initial hospital encounter.^16^

Taken together, our results and other prior studies on patients leaving AMA suggest several tailored interventions that should be studied as potential approaches toward reducing readmissions among this population. During the inpatient hospitalization, clear patient-centered communication to convey the severity of illness and rationale for treatment may compel patients to follow through with their care.^5,17^ After discharge, creating access to drop-in substance use treatment or mental health counseling, arranging primary care follow-up in the first few days after discharge to prevent bounce back readmissions, and frequent check-ins (eg, via home health aide visits and telephone encounters) to prevent these patients from being lost to follow-up may help ensure continuity of care for this population.^6,7,18,19,20^

### Limitations

These findings must be interpreted in the context of our study design. As with all readmission studies, it is difficult to determine the relatedness of readmissions after an index admission. We used temporality as a proxy for relatedness, defining readmissions as any hospitalization starting within 30 days of an index discharge.^21^ This approach also reflects how the Medicare Readmissions Reduction Program calculates penalties, which do not distinguish between readmission diagnoses when determining a hospital’s readmission rate.^22^ Notably, because many patients who leave AMA do so repeatedly, our methods counted subsequent admissions that occurred more than 30 days after initial discharge as unique and separate index admissions to capture this behavior.

In addition, this data set only reports in-hospital mortality, which may significantly preferentially underestimate mortality for the patient population that leaves AMA. A 30-day window to capture mortality rate may also be insufficient because other studies have reported 90-day or even 12-month mortality rates for AMA patients.^3^ The NRD is limited to 1 year of historical discharge data, and patient linkage numbers do not track across years, so follow-up time for patient outcomes after index discharge is limited.

Our estimation of total hospital use and costs attributable to 30-day readmissions after AMA discharge may overstate the overall financial effect of readmissions for these patients because there would have also been an additional cost had these patients not left AMA and completed the full duration of their index admission. However, we cannot extrapolate what the full cost of a completed index admission would have been, nor can we determine whether that additional cost equals the cost of additional readmissions for this patient population.

As with all large, anonymized administrative databases, severity of clinical diagnoses is not captured, which may account for some variation in readmission rates. Further, readmission risk is influenced by factors such as socioeconomic and educational status and community and support systems that are not fully captured in this database; such unadjusted confounders may bias our results. These results are also not generalizable to federal institutions (eg, Veterans Health Administration hospitals) because these data are excluded from the analyses.

## Conclusions

In this nationally representative patient population, individuals discharged AMA had higher 30-day readmission and lower in-hospital mortality rates when compared with all others, at a tremendous cost to the health care system. Patients discharged AMA are also more likely to be readmitted for mental health and substance use disorders, to be readmitted to different hospitals, and to have earlier readmissions after initial discharge. Tailored interventions that address the particular challenges that patients leaving AMA face, such as communication barriers, mental health/substance use comorbidities, and lack of established primary care, should be considered to improve outcomes after initial hospital discharge. Further research is needed to determine whether risk stratification can identify individuals leaving AMA at highest risk for readmission and which of these multilevel efforts may improve health outcomes for this vulnerable patient population.

## Notes

Supplement. **eTable 1. **Top 10 weighted readmission primary diagnoses for AMA vs non-AMA discharges **eTable 2. **Top 10 weighted index admission primary diagnoses for AMA vs non-AMA discharges
