# Supplementary material for: Association of Hospital Discharge Against Medical Advice With Readmission and In-Hospital Mortality
Source: JAMA Netw Open. 2020 Jun 11;3(6):e206009. doi: 10.1001/jamanetworkopen.2020.6009 (PMC7290410; doi:10.1001/jamanetworkopen.2020.6009)
Supplement: Supplement. — eTable 1. Top 10 weighted readmission primary diagnoses for AMA vs non-AMA discharges eTable 2. Top 10 weighted index admission primary diagnoses for AMA vs non-AMA discharges [file jamanetwopen-3-e206009-s001.pdf]

Supplementary Online Content

Tan SY, Feng JY, Joyce C, Fisher J, Mostaghimi A. Association of hospital discharge against medical advice with readmission and in-hospital mortality. *JAMA Netw Open*. 2020;3(6):e206009. doi:10.1001/jamanetworkopen.2020.6009

eTable 1. Top 10 weighted readmission primary diagnoses for AMA vs non-AMA discharges
eTable 2. Top 10 weighted index admission primary diagnoses for AMA vs non-AMA discharges

This supplementary material has been provided by the authors to give readers additional information about their work.

© 2020 Tan SY et al. *JAMA Network Open.*

Supplemental Table 1: Top 10 weighted readmission primary diagnoses for AMA versus non-AMA discharges[1]

**Readmissions with AMA as index disposition**		**Readmissions with all other index disposition**	
Readmission diagnosis	*% of readmissions (95% CI)*	*Readmission diagnosis*	*% of readmissions (95% CI)*
1) Diseases of the heart	12.0 (11.5 – 12.5)	1) Diseases of the heart	13.6 (13.4 – 13.8)
2) Alcohol-related disorders	9.4 (8.3 – 10.5)	2) Bacterial infection	8.3 (8.1 – 8.5)
3) Diabetes mellitus with complications	5.2 (4.9 – 5.5)	3) Non-specific complications[1]	8.2 (7.9 – 8.4)
4) Bacterial infection	5.0 (4.7 – 5.3)	4) Diseases of the urinary system	5.8 (5.7 – 5.9)
5) Skin and subcutaneous tissue infections	4.3 (4.0 – 4.6)	5) Respiratory infections	3.8 (3.7 – 3.9)
6) Mood disorders	4.2 (3.8 – 4.6)	6) Lower gastrointestinal disorders	3.1 (3.1 – 3.2)
7) Substance-related disorders	3.7 (2.8 – 4.5)	7) Mood disorders	3.0 (2.8 – 3.2)
8) Non-specific complications[2]	3.1 (2.9 – 3.4)	8) COPD and bronchiectasis	2.6 (2.5 – 2.6)
9) Diseases of the urinary system	3.0 (2.7 – 3.2)	9) Respiratory failure; insufficiency; arrest	2.5 (2.4 – 2.6)
10) Pancreatic disorders (not diabetes)	2.9 (2.7 – 3.2)	10) Diabetes mellitus with complications	2.4 (2.3 – 2.4)

[1] Primary readmission diagnoses categorized by multi-level CCS hierarchical groupings

[2] Non-specific complications include: complications of implant, graft and device; complications of surgical/medical treatments

© 2020 Tan SY et al. *JAMA Network Open.*

Supplemental Table 2: Top 10 weighted index admission primary diagnoses for AMA versus non-AMA discharges[1]

**Index admissions resulting in AMA disposition**		**Index admissions with all non-AMA dispositions**	
Index admission diagnosis	*% of admissions (95% CI)*	*Index admission diagnosis*	*% of admissions (95% CI)*
1) Diseases of the heart	11.3 (10.8 – 11.7)	1) Diseases of the heart	13.0 (12.7 – 13.2)
2) Alcohol-related disorders	8.8 (7.9 – 9.8)	2) Non-traumatic joint disorders	5.4 (5.0 – 5.7)
3) Substance-related disorders	7.1 (5.4 – 8.8)	3) Bacterial infection	5.1 (5.0 – 5.3)
4) Skin and subcutaneous tissue infections	4.6 (4.4 – 4.9)	4) Diseases of the urinary system	4.7 (4.6 – 4.8)
5) Bacterial infection	4.2 (4.0 – 4.5)	5) Lower gastrointestinal disorders	4.0 (3.9 – 4.0)
6) Diabetes mellitus with complications	3.9 (3.8 – 4.1)	6) Fractures	3.9 (3.7 – 4.0)
7) Symptoms, signs and ill-defined conditions	3.6 (3.4 – 3.8)	7) Respiratory infections	3.7 (3.6 – 3.7)
8) Diseases of the urinary system	3.4 (3.3 – 3.6)	8) Complications[2]	3.6 (3.5 – 3.7)
9) Respiratory infections	3.0 (2.9 – 3.2)	9) Cerebrovascular disease	3.5 (3.4 – 3.6)
10) Mood disorders	2.9 (2.4 – 3.4)	10) Mood disorders	2.9 (2.6 – 3.1)

[1] Primary readmission diagnoses categorized by multi-level CCS hierarchical groupings

[2] Non-specific complications include: complications of implant, graft and device; complications of surgical/medical treatments

© 2020 Tan SY et al. *JAMA Network Open.*
